# Spinocerebellar Ataxias: Phenotypic Spectrum of PolyQ *versus* Non-Repeat Expansion Forms

**DOI:** 10.1007/s12311-024-01723-9

**Published:** 2024-07-24

**Authors:** João Moura, Jorge Oliveira, Mariana Santos, Sara Costa, Lénia Silva, Carolina Lemos, José Barros, Jorge Sequeiros, Joana Damásio

**Affiliations:** 1 Neurology Department, Centro Hospitalar Universitário de Santo António, ULS de Santo António, Porto, Portugal; 2 Centro de Genética Preditiva e Preventiva (CGPP), IBMC - Institute for Molecular and Cell Biology, Universidade do Porto, Porto, Portugal; 3 IBMC - Institute for Molecular and Cell Biology, i3S - Instituto de Investigação e Inovação em Saúde, Universidade do Porto, Porto, Portugal; 4 ICBAS School of Medicine and Biomedical Sciences, Universidade do Porto, Porto, Portugal

**Keywords:** Spinocerebellar Diseases, Hereditary Ataxia, Hereditary Spinocerebellar Degenerations, Trinucleotide Repeat Expansions, Machado-Joseph disease

## Abstract

Spinocerebellar ataxias (SCA) are most frequently due to (CAG)_n_ (coding for polyglutamine, polyQ) expansions and, less so, to expansion of other oligonucleotide repeats (non-polyQ) or other type of variants (non-repeat expansion SCA). In this study we compared polyQ and non-repeat expansion SCA, in a cohort of patients with hereditary ataxia followed at a tertiary hospital. From a prospective study, 88 patients (51 families) with SCA were selected, 74 (40 families) of whom genetically diagnosed. Thirty-eight patients (51.4%, 19 families) were confirmed as having a polyQ (no other repeat-expansions were identified) and 36 (48.6%, 21 families) a non-repeat expansion SCA. Median age-at-onset was 39.5 [30.0-45.5] for polyQ and 7.0 years [1.00-21.50] for non-repeat expansion SCA. PolyQ SCA were associated with cerebellar onset, and non-repeat expansion forms with non-cerebellar onset. Time to diagnosis was longer for non-repeat expansion SCA. The most common polyQ SCA were Machado-Joseph disease (MJD/SCA3) (73.7%) and SCA2 (15.8%); whereas in non-repeat expansion SCA ATX-*CACNA1A* (14.3%), *ATP1A3*-related ataxia, ATX-*ITPR1*, ATX/HSP-*KCNA2,* and ATX-*PRKCG* (9.5% each) predominated. Disease duration (up to inclusion) was significantly higher in non-repeat expansion SCA, but the difference in SARA score was not statistically significant. Cerebellar peduncles and pons atrophy were more common in polyQ ataxias, as was axonal neuropathy. SCA had a wide range of genetic etiology, age-at-onset and presentation. Proportion of polyQ and non-repeat expansion SCA was similar; the latter had a higher genetic heterogeneity. While polyQ ataxias were typically linked to cerebellar onset in adulthood, non-repeat expansion forms associated with early onset and non-cerebellar presentations.

**Supplementary Information:**

The online version contains supplementary material available at 10.1007/s12311-024-01723-9.

## Introduction

Spinocerebellar ataxias (SCA) comprise a heterogeneous group of rare autosomal dominant diseases, characterized by ataxia as the cardinal symptom, but showing a wide neurological and extra-neurological expression [1, 2]. Age-at-onset (AO) is commonly between the fourth and sixth decades, with pediatric and later-onset forms being classically considered as less frequent [3–5]. Inter and intra-familial phenotypic diversity increases its diagnostic difficulties [6].

The most frequent SCA worldwide are those caused by CAG repeat expansions, as SCA1 (in *ATXN1*), DRPLA (*ATN1*)*,* SCA2 (*ATXN2*), Machado-Joseph disease (MJD)/ SCA3 (*ATXN3*), SCA6 (*CACNA1A*), SCA7 (*ATXN7),* and SCA17 (*TBP*), usually designated polyglutamine (polyQ) ataxias [7, 8]. As in other polyQ diseases, an inverse correlation is seen between AO and size of the expanded (CAG)_n_, longer repeats being associated with earlier onset and a more severe phenotype [9, 10]. Expansion of non-CAG repeats and non-repeat expansion SCA (caused by conventional variants as single-nucleotide substitutions or small insertions/deletions) are classically considered rare; nevertheless, its recognition has increased in past years, mostly due to the routine application of next generation sequencing (NGS)-based genetic studies [9, 11, 12]. As opposed to polyQ, non-repeat expansion SCA usually present during childhood, show no anticipation of AO, progress more slowly and may have congenital features (as psychomotor delay) [11, 12]. Studies comparing these two groups of SCA are scarce.

In this report, we compare the clinical and genetic features of polyQ *versus* non-repeat expansion SCA, in a cohort of hereditary cerebellar ataxias (HCA) followed at a tertiary center in the North of Portugal.

## Patients and Methods

### Patient Selection

A prospective study on HCA has been conducted at ULSSA, since 2017. Patients with dominant HCA (SCA) were selected from the local ataxia database. From 249 patients (164 families) with HCA, 88 (51 families, 31.1%) had SCA; of these, 74 (40 families, 78.4%) had an established genetic diagnosis, but 11 families were still at different stages of investigation.

All 249 patients were evaluated through a structured protocol (listed in Suppl. Material), comprising a baseline detailed identification of AO of different neurological symptoms/signs (e.g., dysarthria, dysphagia, limbs’ coordination, epilepsy, pyramidal signs, movement disorders), and motor disability milestones (agraphia, falls, gait assistance, confinement to wheelchair). Every twelve months, a questionnaire on new neurological symptoms and disability was applied. The Scale for the Assessment and Rating of Ataxia (SARA) and Inventory of Non-Ataxia Signs (INAS) were evaluated at baseline and on annual examinations.

### Definitions

*Age-at-onset* (AO) was considered as the age at first neurological symptoms, either cerebellar or non-cerebellar. Whenever patients had been prospectively followed since the presymptomatic stage, AO was considered as the age at which the first symptoms were registered during the study. In patients whose symptoms started before, information collected at the study baseline was checked (whenever possible) with two first-degree relatives and clinical records. In case of discordance, due to memory biases or other reasons, information from clinical records was preferred.

*Epilepsy* was considered only when the participant suffered two or more unprovoked seizures. *Pyramidal signs* were hyperreflexia, spasticity, Hoffman sign, and/or extensor plantar reflex. *Movement disorders* were classified into hypokinetic (Parkinsonism) and hyperkinetic (tremor, dystonia, chorea, myoclonus, tics). *Motor delay* was defined when motor milestones were outside the 95% confidence interval, according to the World Health Organization [13]. *Intellectual disability* was defined as intellectual difficulties, but also problems in conceptual, social, and practical areas of living, according to the Diagnostic and Statistical Manual of Mental Disorders, 5^th^ edition [14]. *Hemiplegic migraine* was considered in presence of migraine with aura, including fully reversible motor weakness and fully reversible visual, sensory, and/ or speech/ language symptoms. *Dysphagia* was registered when occurring repeatedly and leading to bursts of cough. *Psychiatric symptoms* (anxiety, depression, psychosis) were diagnosed as *per* clinical judgement. *Motor disability milestones* included agraphia, falls, use of cane, or confinement to wheelchair. *Agraphia* was considered when the patients could no longer sign their name. *Falls* were registered only if occurring repeatedly. *Loss of independent gait* corresponded to the use of at least one cane; *confinement to wheelchair* meant the permanent use of this aid.

### Sample Characterization

Patients selected, with SCA, were classified according to the revised nomenclature on Genetic Movement Disorders (except for SCA1, SCA2, MJD/SCA3, SCA6, and SCA7, more readily recognized by their original designations) [15]. For genes not yet associated with an ataxic phenotype by the International Parkinson and Movement Disorder Society Task Force on Classification and Nomenclature of Genetic Movement Disorders, we used the suffix “-related ataxia” after the gene designation. Sex, AO, age at observation, and at genetic diagnosis was recorded. Presenting clinical features, associated neurological symptoms and SARA score were collected. In all patients with disease onset in the first decade of life, metabolic disorders had been excluded *ab initium*. Age, cause and place of death were documented, according to the medical records, if available, or as stated by first degree relatives. Brain MRI was initially performed with 1.5 or 3.0 tesla scanners. Electromyography (EMG) was conducted according to clinical judgement.

Analyses of AO, additional symptoms/ signs, and disability milestones were performed. Brain MRI and EMG were also considered for analysis.

### Genetic Testing

**Fig. 1:**
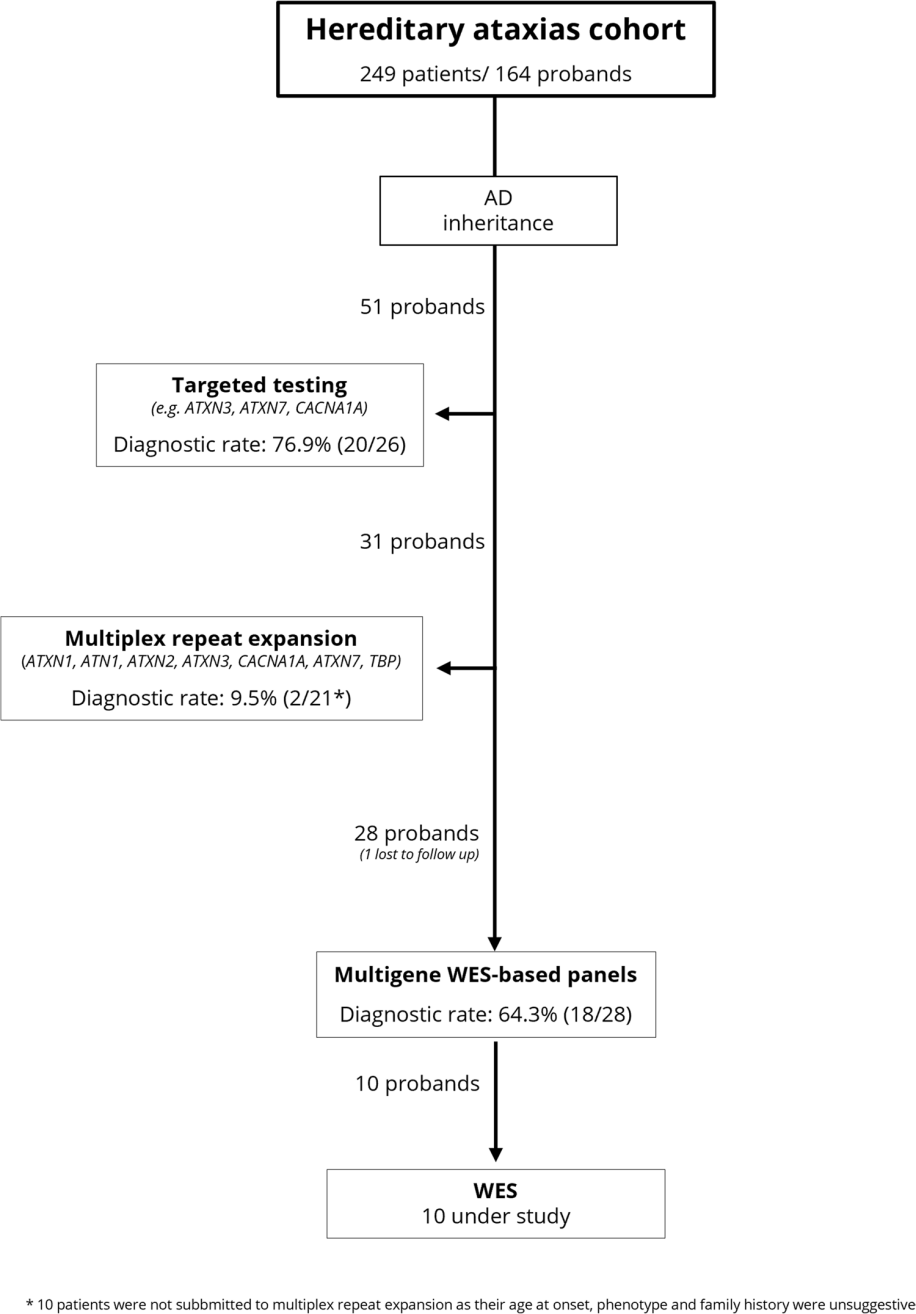
Flowchart of genetic diagnostic strategy and diagnostic yield

DNA was collected from peripheral blood and stored at CGPP, IBMC. Following the variants’ classification guidelines of American College of Medical Genetics and Genomics[16], a genetic diagnosis was considered as achieved in the presence of one pathogenic/ likely pathogenic variant in heterozygosity, in a gene associated with a dominant phenotype. The *diagnostic yield* (or detection rate) was defined here as the number of patients in whom a genetic diagnosis was established, divided by the total number of patients submitted to that assay (Fig. 1). A proband was, by definition, the first patient to be genetically diagnosed in the family. For the 51 probands, targeted (single-gene) testing was performed in 26, in whom a specific form of SCA was highly suspected (e.g., MJD/SCA3 due to its high prevalence in geographical clusters, SCA7 in the presence of retinopathy, *CACNA1A* expansion in case of a pure cerebellar syndrome, and *CACNA1A* conventional variants in familial hemiplegic migraine) – the diagnostic yield was 76.9% (20/26). In 21 probands (6 undiagnosed after targeted testing, and 25 not submitted to targeted testing – details in Suppl. Material) testing for expansions of the CAG repeat in *ATXN1, ATN1, ATXN2, ATXN3, CACNA1A, ATXN7* and *TBP*, through multiplex polymerase chain reaction (PCR) and fragment analysis was performed – a diagnostic yield of 9.5% (2/21). Ten probands were not submitted to genetic study of polyQ ataxia as their age at onset, clinical presentation and/or family history were highly unsuggestive (details in Suppl. Material). Finally, out of 29 probands, one was lost to follow up and 28 were studied through multigene panels (genes detailed in Suppl. Material) based on whole-exome sequencing (WES) – diagnostic yield of 64.3% (18/28). Ten probands are under WES study. In affected relatives of probands, diagnosis was by single-gene testing. Variants were considered *de novo* only if both parents had been genotyped and were not carriers.

### Statistical Analysis

For descriptive statistics, qualitative variables were studied through absolute and relative frequency. Mean with standard deviation or median with interquartile ranges (IQR [Q1-Q3]) were used for quantitative variables, depending on variables following or not the Normal distribution. Group comparisons were performed using the chi-square test for qualitative variables; for small sized samples, the Monte Carlo method was adopted. For quantitative variables, the Student’s t (in Normal) or Mann-Whitney tests were used (in non-Normal distribution). The Spearman test was used to assess a possible correlation between number of repeats and AO. A binary logistic regression model was created to evaluate whether every SCA type associated with a particular disability milestone, controlling for disease duration, as in previous studies.[5, 17, 18] A Hosmer-Lemeshow test was used to test fitness of the model (*p*<0.05). Statistical analysis was done using the IBM Statistical Package for the Social Sciences (SPSS), version 27. A *P* value < 0.05 was considered as statistically significant.

### Ethical Considerations

The project was approved by the ULSSA Ethics Committee. Informed consent was obtained in all patients for genetic testing and clinical investigation.

## Results

Among the patients with genetic diagnosis, 38 (19 families, 47.5%) had polyQ and 36 (21 families, 52.5%) had non-repeat expansion SCA. Table 1 summarizes the demographic and clinical features between both groups. Of the 11 SCA probands without genetic diagnosis (excluded from the clinical analysis), 10 were being studied through WES and 1 was lost to follow up (studied with PCR and fragment analysis).

**Table 1:** Demographic and clinical data for polyglutamine and non-repeat expansion ataxias

	Polyglutamine ataxias	Non-repeat expansion ataxias	*P* value
Number of probands	19 *(47.5%)*	21 (*52.5%*)	
Total number of patients	38 (*51.4%*)	36 (*48.6%*)	
Female gender	18 (*47.4%*)	24 (*66.7%*)	0.107
Age at onset*	39.5 y *[30.0-45.5]*	7.0 y [*1.0-21.5*]	**<0.001**
Age at diagnosis*	41.0 y [*33.8-53.0*]	37.5 y [*24.8-48.3*]	0.1
Time to diagnosis*	4.0 y [*1.0-9.0*]	22.0 y [*15.0-36.3*]	**<0.001**
Disease duration until baseline*	10.0 y [*4.7-18.7*]	26.0 y [*17.0-34.7*]	**<0.001**
Type of onset:			
Cerebellar	35 (*92.1%*)	17 (*47.2%*)	**<0.001**
Non-cerebellar	3 (*7.9%*)	19 (*52.8%*)	
Detailed symptoms at disease onset:			NA
Cerebellar:			
Gait unsteadiness	28 (*73.7%*)	11 (*30.6%*)	
Upper limb dysmetria	3 (*7.9%*)	3 (*8.3%*)	
Dysarthria	1 (*2.6%*)	-	
Diplopia	3 (*7.9%*)	-	
Non-cerebellar:			
Motor delay	-	8 (*22.2%*)	
Hemiplegic migraine	-	6 (*16.7%*)	
Paroxystic ataxia	-	3 (*8.3%*)	
Parkinsonism	2 (*5.3%*)	-	
Seizures	-	2 (*5.6%*)	
Chorea	-	1 (*2.8%*)	
Paroxystic dystonia		1 (*2.8%*)	
Retinopathy	1 (*2.6%*)	-	
Spastic gait	-	1 (*2.8%*)	
Precipitating events for disease onset	5 (*13.1%*)	11 (*30.5%*)	0.051
SARA score at baseline	13.0 [*6.5-22.1*]	14.0 [*9.0-15.0*]	0.785
Symptoms throughout disease course:			
Gait unsteadiness	38 (*100.0%*)	36 (*100.0%*)	0.243
Dysmetria (upper and lower limb)	38 (*100.0%*)	35 (*97.2%*)	0.618
Dysarthria	30 (*78.9%*)	34 (*94.4%*)	**0.015**
Dysphagia	31 (*75.6%*)	17 (*47.2%*)	**0.018**
Ocular signs	35 (*92.1%*)	26 (*72.2%*)	**0.033**
Diplopia	26 (*68.4%*)	4 (*11.1%*)	**<0.001**
Nystagmus	22 (*57.9%*)	13 (*36.1%*)	0.169
Horizontal ophthalmoparesis	24 (*63.2%*)	4 (*11.1%*)	**<0.001**
Vertical ophthalmoparesis	18 (*47.4%*)	4 (*11.1%*)	**0.002**
Hypermetric saccades	22 (*57.9%*)	14 (*38.9%*)	0.254
Hypometric saccades	11 (*28.9%*)	9 (*25.0%*)	1.000
Oculomotor apraxia	2 (*5.3%*)	2 (*5.6%*)	1.000
Pyramidal signs	23 (60.5*%*)	21 (*58.3%*)	0.848
Intellectual disability	-	11 (*30.6%*)	**<0.001**
Dementia	5 (*13.2%*)	5 (*13.9%*)	1.000
Epilepsy	-	5 (*13.9%*)	**0.023**
Movement disorders	28 (*73.7%*)	24 (*66.7%*)	0.613
Dystonia	27 (*71.1%*)	22 (*61.1%*)	0.463
Tremor	7 (*18.4%*)	4 (*11.1%*)	0.517
Chorea	2 (*5.3%*)**	5 (*13.9%*)	0.255
Parkinsonism	4 (*10.5%*)	1 (*2.8%*)	0.358
Myoclonus	-	3 (*8.3%*)	0.110
Peripheral signs	22 (*57.9%*)	6 (*16.7%*)	**<0.001**
Neuropathy	19 (*50.0%*)	6 (*16.7%*)	**0.003**
Muscle cramps	9 (*23.7%*)	-	**0.002**
Psychiatric comorbidities	29 (*76.3%*)	13 (*37.1%*)	**<0.001**
Depression	26 (*68.4%*)	10 (*28.6%*)	**<0.001**
Anxiety	24 (*63.2%*)	12 (*34.3%*)	**0.019**
Psychosis	0 (*0.0%*)	1 (*2.9%*)	0.479
Disability milestones			
Agraphia	7 (*17.1%*)	2 (*5.6%*)	0.162
Falls	29 (*70.7%*)	14 (*38.9%*)	**0.006**
Unilateral assistance in gait	18 (*43.9%*)	10 (*27.8%*)	0.162
Wheelchair bound	13 (*31.7%*)	2 (*5.6%*)	**0.004**

Legend:* NA* Not applicable, *Y* Years. *Age expressed in mean and interquartile range [Q1-Q3], ** Two patients with concurrent Huntington disease

### Genetic Diagnosis

**Fig. 2:**
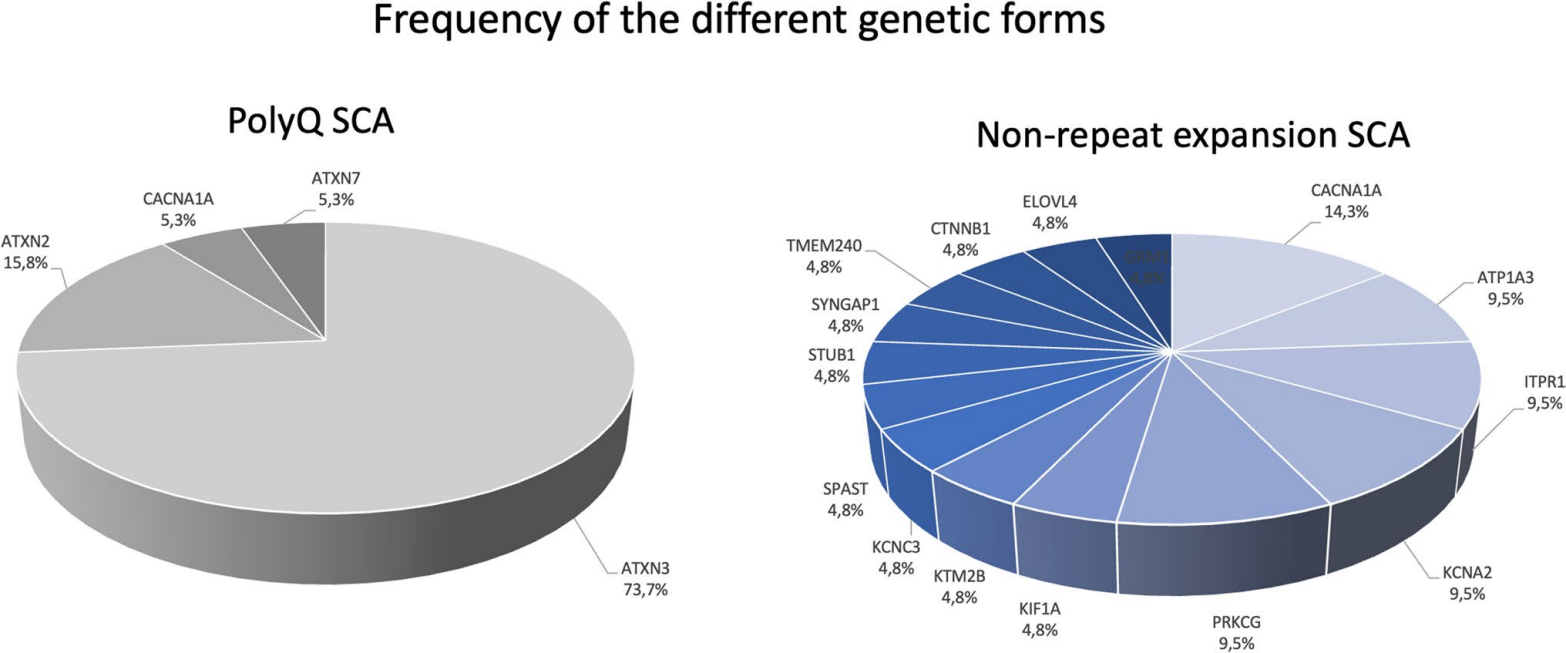
Frequency of the various genetic forms in polyQ and non-repeat expansion SCA

Diagnosis in polyQ SCA probands was achieved through targeted testing in 17 (89.5%), and multiplex approach in 2 (10.5%). In non-repeat expansion SCA, diagnosis in a proband was obtained by targeted testing in 3 (14.3%) and NGS-based panels in 18 (85.7%). Median time to diagnosis was 4.0 [1.0-8.0] years for polyQ and 25.0 [17.0-33.5] for non-repeat expansion SCA (*p*<0.001). The most common polyQ form was MJD/SCA3, in 14 (73.7%) families; followed by SCA2, in 3 (15.8%); and SCA7 and SCA6, in one (5.3%) family each (Fig. 2). Mean number of repeats in expanded and normal alleles for polyQ is presented in Suppl. Table 1. Genetic diagnoses were more diverse in non-repeat expansion SCA, with pathogenic variants involving 17 genes, the most frequent being ATX-*CACNA1A* (3 families, 14.3%) and *ATP1A3*-related ataxia, ATX-*ITPR1*, ATX/HSP-*KCNA2,* and ATX-*PRKCG* (2 families, 9.5% each) (Fig. 2). *De novo* mutations were seen only in non-repeat expansion forms (8 cases, 38.1% of tested families). Detailed number of patients per families is shown in Suppl. Tables 2 and 3.

### Age-at-Onset and Presenting Symptoms

**Fig. 3:**
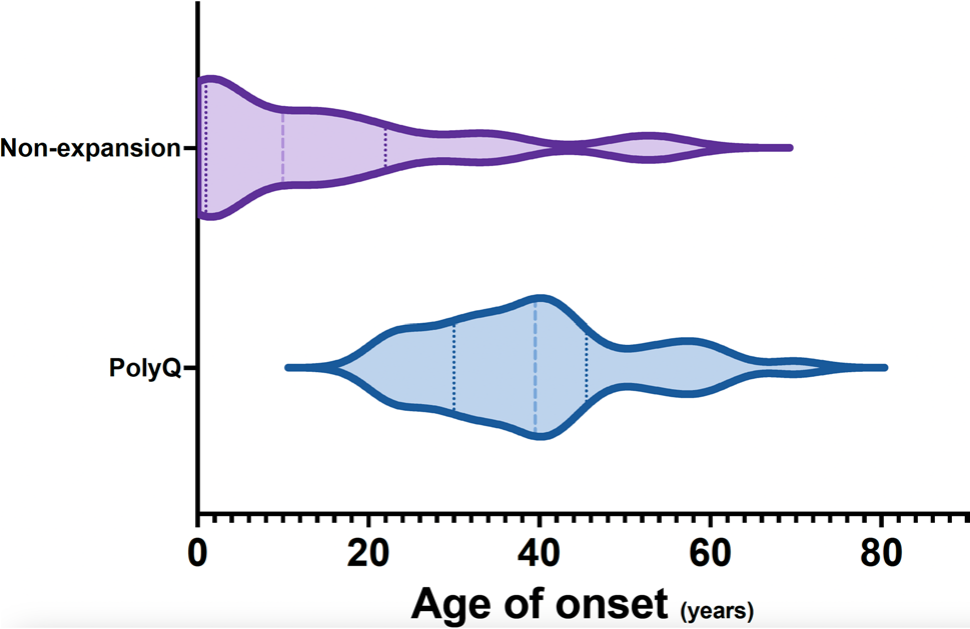
Distribution of age-at-onset in polyQ and non-repeat expansion SCA

Considering all 74 patients with genetic diagnosis (probands and observed affected relatives, Suppl. Table 2), median AO was 39.5 [30.0-45.5] in polyQ and 7.0 years [1.0-21.5] in non-repeat expansion SCA (*p*<0.001). Patients in the non-repeat expansion group had a wider range of AO, from congenital up to age 55 years (Fig. 3). In MJD/SCA3, there was an inverse correlation between size of the large allele and AO (Spearman rho=-0.47, 95% CI:0.75,-0.05, *p*=0.027). The same was seen in SCA2, though only marginally significant, probably due to the low number of patients (Spearman rho=-0.67, CI:0.92,-0.04, *p*=0.055).

Cerebellar onset was significantly associated with polyQ, and non-cerebellar onset with non-repeat expansion SCA (*p*<0.001). The most frequent presenting symptom was gait unsteadiness in both groups: 28 (73.7%) in polyQ, and 11 (30.6%) in non-repeat expansion SCA. PolyQ ataxias presented predominantly (35, 92.1%) with cerebellar signs (unsteady gait, diplopia, dysarthria, dysmetria); two patients (4.8%) with MJD/SCA3 had onset with Parkinsonism, and one (2.4%) SCA7 with retinopathy. Non-repeat expansion SCA had a wider range of presenting symptoms, particularly non-cerebellar features: motor delay in congenital forms (8, 22.2%), paroxystic ataxia (3 cases, 8.3%), hemiplegic migraine (6, 16.7%), seizures (2, 5.6%), and other (less frequent), as detailed in Table 1. Precipitating events for disease onset were suspected in 5 (13.1%) polyQ and 11 (30.5%) non-repeat expansion forms; the most common were pregnancy (7, 43.8%) and infection (4, 25.0%).

### Disease Progression and Disability Milestones

Median duration from disease onset up to inclusion was 10 [4.8-18.8) years for polyQ and 26.0 [17.0-34.8] for non-repeat expansion SCA (*p*<0.001), with median prospective follow-up being of 4.0 [2.0-5.0] and 4.5 [2.0-5.0]. Even though disease duration until baseline was significantly higher for non-repeat expansion SCA, there was no significant difference in total SARA score at baseline (13.0 [6.5-22.1] for polyQ and 14.0 [9.0-15.0] for non-repeat expansion). Symptoms throughout disease course also differed between groups (Table 1 and Suppl. Tables 3 and 4): polyQ SCA were more frequently associated with dysphagia (*p*=0.018), ocular signs (*p*=0.033), peripheral signs (*p*<0.001), and psychiatric manifestations (*p*<0.001); non-repeat expansion SCA displayed more commonly dysarthria (*p*=0.015), intellectual disability (*p*<0.001), and epilepsy (*p*=0.023). There was no significant difference in non-ataxia movement disorders associated; the only two patients with chorea had concurrent Huntington disease. Diplopia (*p*=0.010), dysphagia (*p*<0.001), and gait unsteadiness (*p*<0.001) occurred after a shorter disease duration in polyQ ataxias (Suppl. Table 4). Considering disability, a higher frequency of falls (70.7% *vs*. 38.9%, *p*=0.006) and confinement to wheelchair (31.7% *vs.* 5.6%, *p*=0.004) was seen in polyQ-SCA. Disease duration until each disability milestone was similar between groups (Suppl. Table 5), except for assistance in gait (*p*<0.001) which occurred earlier in polyQ. Five (6.5%) patients died throughout the study course, all with MJD/SCA3, at a median age of 76.0 [69.0-78.0] years. Infection (four respiratory, one sepsis) was the cause of death in all. Three died in the hospital, one in a nursing home, and the other at home.

### Complementary Exams

Overall, polyQ had a shorter disease duration until the last brain MRI and EMG. Twenty-eight (73.7%) patients with polyQ and 34 (94.4%) with non-repeat expansion forms had information on brain pattern of atrophy on MRI (Table 2); the only significantly different topographies between groups were atrophy of the pons (*p*=0.027) and cerebellar peduncles (*p*=0.009), more common in polyQ ataxias. EMG was performed in 21 (55.3%) polyQ and 19 (52.8%) non-repeat expansion SCA patients, the first being associated with sensitive axonal neuropathy (*p*=0.006).

**Table 2:** Genetic and complementary investigation of polyglutamine and non-repeat expansion ataxias

	Polyglutamine ataxias	Non-repeat expansion ataxias	*p* value
Number of probands	19 (*47.5%*)	21 (*52.5%*)	
Total number of patients	38 (*51.4%*)	36 (*48.6%*)	
Genetic testing			NA
Targeted test	17 (*89.5%*)	3 (*14.3%*)	
Multiplex PCR and fragment analysis	2 (*10.5%*)	-	
Multigene panel (WES-based)	-	18 (85.7*%*)	
WES	-	-	
*De novo* variant	0	8 (38.1*%*)	**<0.001**
Brain MRI	28 (*73.7%*)	34 (*94.4%*)	**0.015**
Disease duration until last exam	6.0 [*3.0-14.0*]	24.0 [*14.0-37.0*]	**<0.001**
Atrophy			
Vermis	22 (*78.6%*)	24 (*70.6%*)	0.566
Cerebellar hemispheres	21 (*75.0%*)	23 (*67.6%*)	0.584
Cerebellar peduncles	9 (*32.1%*)	2 (*5.9%*)	**0.009**
Pons	9 (*32.1%*)	3 (*8.8%*)	**0.027**
Midbrain	5 (*17.9%*)	1 (*3.0%*)	0.085
Medulla	4 (*14.3%*)	1 (*2.9%*)	0.166
Cortex	4 (*14.3%*)	3 (*8.8%*)	0.691
Nerve conduction studies	21 (*55.3%*)	19 (*52.8%*)	1.000
Disease duration until last exam	10.5 y [*6.8-23.0*]	23.0 y [*16.0-37.0*]	**0.010**
Demyelinating neuropathy	0	0	n.a.
Axonal neuropathy	16 (*72.7%*)	5 (*26.3%*)	**0.005**
Sensitive	16 (*72.7%*)	5 (*26.3%*)	**0.005**
Motor	5 (*22.7%*)	2 (*10.5%*)	0.419

Legend: *NA *Not applicable,* PolyQ* Polyglutamine, *Y* Years, *WES* Whole-exome sequencing

## Discussion

We studied a group of patients with SCA from the North of Portugal. We identified a comparable number of families with polyQ and non-repeat expansion SCA, which was unexpected, as the latter are considered to be responsible for only about 10%-18% of SCA [19, 20]. This could be explained by our cohort having been established at a tertiary referral center, which receives patients from all the northern region of the country, including pediatric neurology clinics (with a higher number of non-repeat expansion SCA). Also, for some time now, the use of NGS has been everyday clinical practice at our center; this has increased the rate of genetic diagnoses in the past years, mostly due to identification of non-repeat expansion forms [11, 12].

### Diagnostic Strategy and Detection Rate

A flowchart for our diagnostic strategy in SCA is illustrated in Fig. 1. A final genetic diagnosis was obtained in 78.4%, at the upper limit of the described (31.3%-89.0%) [20–24]. (1) Single-gene testing was used in a selected group of SCA (owing to a strong clinical suspicion or, particularly for MJD/SCA3, its high prevalence in our country), with a detection rate of 76.9%; the remaining probands, followed established diagnostic recommendations [25]. (2) The diagnostic yield of multiplex PCR and fragment analysis for the most frequent SCA was of only 9.5%, lower than in other series (13.8%-70.8%) [21, 23, 24], as most polyQ probands had already been diagnosed through targeted testing. (3) WES-based multigene panels, had an overall yield of 64.3%, concordant with other series reporting 12.1%-64.4% [19, 21–24, 26]. Ten patients are under (4) WES study, and no cases have yet been summitted to (5) whole-genome sequencing (WGS).

### Relative Frequency of the Specific Genetic Forms

PolyQ were genetically more homogeneous than non-repeat expansion SCA. As in previous studies [7, 27], MJD/SCA3 was, overall, the most frequent form. SCA2 was the second most common polyQ ataxia, replacing dentatorubral-pallidoluysian atrophy (DRPLA), as found earlier in the nation-wide survey [27]. This is most probably explained by the clustering of DRPLA families in the Center and South [28], while our cohort was established in the North. SCA7 and SCA6 were less frequent; both are known to have a national prevalence under 0.10 per 100 000, being also rarer in other European countries [27, 29–32]. As expected [33–35], an inverse correlation of repeat size with AO was observed for MJD/SCA3 and SCA2 (in the remaining polyQ SCA, the low number of patients precluded analysis).

The gene most frequently involved in non-repeat expansion SCA was *CACNA1A*, in line with data from the national survey [27]. *ATP1A3-*related ataxia, ATX-*KCNC3,* and ATX-*ITPR1* had not yet been described by that time; many families had already been identified, but had no definite diagnosis by then. The remaining, ultra-rare, non-repeat expansion forms were present only in one or two families. Relative frequency of specific forms within non-repeat expansion SCA was in accordance with a large international collaborative cohort, where *CACNA1A, PRKCG, AFG3L2, ITPR1, STUB1, SPTBN2*, or *KCNC3* were the most frequent genes [11]. *De novo* pathogenic variants were exclusively identified among non-repeat expansion ataxias (38.1%), all patients with no family history.

### Onset Age and Symptoms

Non-repeat expansion presented earlier than polyQ SCA (7.0 *vs.* 39.5 years), consistent with previous findings. AO in polyQ ataxias is usually between 30 and 50 years of age [4, 5, 34, 36], while in channelopathies and other non-repeat expansion SCA tends to occur sooner [12, 19]. Still, as in other cohorts, we found a wide range of AO in non-repeat expansion SCA, spanning from congenital up to onset in the sixth decade of life [11, 24].

Clinical spectrum differed significantly between groups. In polyQ SCA, cerebellar onset was patent in the majority; whereas in non-repeat expansion SCA, onset with cerebellar and non-cerebellar signs was similarly frequent. Congenital forms and paroxystic episodes (seizures, hemiplegic migraine, ataxia, dystonia) were exclusively seen with non-repeat expansions, as in previous descriptions [11, 19, 32]. The increased occurrence of paroxystic episodes in non-expansion SCA was probably explained by the high frequency of genes (e.g., *CACNA1A, KCNA2, ATP1A3*) coding for ion channels [37–39]. Pathogenic variants in *CACNA1A, KCNA2* and *KMT2B* were associated with epilepsy; seizures are a known feature of the first two [40, 41]. Interestingly, some cases corresponded to channelopathies associated with cognitive and motor delay, in which ataxia has also been described, including *KCNC3, ITPR1, ATP1A3* and *CTNNB1* [42–46].

Over disease course, polyQ ataxias displayed more frequently ocular disturbances (diplopia and ophthalmoparesis) and peripheral signs (neuropathy and muscle cramps), both known to be associated with MJD/SCA3 and SCA2, the most frequent forms [47, 48]. Depression and anxiety, associated with polyQ SCA, occurred at a higher frequency than in general population [49]. Intellectual disability was exclusive for non-repeat expansion SCA, while dementia was present in both groups, in line with other descriptions [9, 11].

### Subsidiary Exams

Atrophy of the pons and cerebellar peduncles on MRI was the distinguishing feature between groups, associating with polyQ SCA. Quantitative brain MRI studies have described atrophy in the cerebellar hemispheres, vermis, midbrain, pons, medulla oblongata and cervical spine in polyQ SCA, particularly in MJD/SCA3 [9, 50]. In non-repeat expansion SCA, cerebellar atrophy with no brainstem involvement is common [9, 51, 52], in line with what was observed in our series. Also, the axonal sensitive neuropathy on EMG, associated with polyQ SCA, is consistent with the clinical picture of MJD/SCA3 [53].

### Disease Progression and Disability

At baseline, there was no significant difference between groups in global SARA score, notwithstanding a significantly shorter disease duration in polyQ ataxias suggesting faster disease progression; inclusion of patients with childhood or juvenile onset of polyQ SCA (not observed in our series) would have made this difference even more marked, as these usually have accelerated progression [9]. PolyQ ataxias significantly associated with falls and wheelchair confinement (Table 1), and earlier assistance in gait (Suppl. Table 5) These findings are in line with the fact that non-repeat expansion SCA have slower disease progression, and use of gait assistance devices is less frequent and later [9, 12]. Knowing clinical progression and disability milestones is of the utmost importance, as they are linked to survival [54, 55].

Life expectancy is reduced in polyQ SCA [54]. All our deceased patients had MJD/SCA3; they lived, on average, 5.6 years less than the general population in northern Portugal [56].

### Limitations of this Study

This is a relatively small sample, from a tertiary hospital receiving complex patients, what prevents generalization of our conclusions. Also, as the study started in 2017, a recall bias of symptoms emerging earlier cannot be excluded. To overcome this, we collected information from patients and relatives, and checked it with their previous clinical records. Our polyQ group included mainly MJD/SCA3 and SCA2, what may have biased analysis of phenotype. Also, as non-repeat expansion SCA consisted of small groups with variable phenotype, this prevented a more detailed analysis and, possibly, establishing stronger associations. Nevertheless, patients with polyQ and non-repeat expansion SCA were in similar number, reinforcing comparative power between them, our main purpose. Imaging was based on retrospective appraisal of MRI; thus, we were unable to explore patterns specific for each SCA.

## Conclusions

This study defined the major clinical features and progression in polyQ and non-repeat expansion SCA. We highlight the earlier onset, congenital forms, and paroxystic symptoms with non-expansion variants; and adult-onset, cerebellar presentation, ocular and peripheral signs with polyQ. Longer time-to-diagnosis in non-repeat expansion SCA probably occurs due to a combination of factors: early-onset, non-cerebellar presentations, slower progression, lesser severity, and *de novo* variants. Increasing recognition (and widespread use of NGS-based tests, including WGS) will favor a timelier diagnosis. We believe these findings may be of value for future research and impact clinical practice of neurologists, pediatricians, and clinical and laboratory genetics.

## Supplementary Information

ESM 1 Supplementary Material Methods: clinical protocol, Workflow of genetic testing, screening of ataxias caused by repeats’ expansions methodology, NGS based panels methodology, list of genes in multigene panels, whole exome sequencing methodology. (DOCX 34 kb)

ESM 2 Supplementary Table 1 Median number of repeats in each allele in patients from the polyQ ataxia group. Supplementary Table 2 Genetic forms in polyQ and non-repeat expansion SCA. Supplementary Table 3 Detailed clinical characterization of patients with polyQ ataxias (individual data). Supplementary Table 4 Detailed clinical characterization of patients with non-repeat expansion SCA (individual data). Supplementary Table 5 Disease duration in years [Q1-Q3] until symptom and disability milestone ultimately reached, according to the type of ataxia (DOCX 56 kb)

## Data Availability

No datasets were generated or analysed during the current study.
