# Supplementary material for: Spinocerebellar Ataxias: Phenotypic Spectrum of PolyQ versus Non-Repeat Expansion Forms
Source: Cerebellum. 2024 Jul 24;23(6):2258–68. doi: 10.1007/s12311-024-01723-9 (PMC11585503; doi:10.1007/s12311-024-01723-9)
Supplement: Supplementary file 1 — Supplementary Material Methods: clinical protocol, Workflow of genetic testing, screening of ataxias caused by repeats’ expansions methodology, NGS based panels methodology, list of genes in multigene panels, whole exome sequencing methodology. (DOCX 34 kb) [file 12311_2024_1723_MOESM1_ESM.docx]

**SUPPLEMENTARY MATERIAL**

**A. Methods**

**1. Clinical protocol**

*Demographic information*: Sex, date of birth, age of symptom onset, age at first observation, age at diagnosis, place of birth, family history, and history of consanguinity.

*Genetic information*: Pathogenic variants, mode of transmission, and genetic test used.

*Neurological symptoms/ signs and age of appearance of each:* nystagmus, dysarthria, dysphagia, diplopia, hyper/ hypometric saccades, dysmetria, gait ataxia, ophthalmoparesis, oculomotor apraxia, pyramidal signs (hyperreflexia in four limbs, spasticity, Babinski and/ or Hoffman signs), dystonia, tremor (rest, postural or kinetic), Parkinsonism, chorea, myoclonus, epilepsy, anxiety, depression, cognitive or motor delay, and hypotonia. Hemiplegic migraine was considered when in presence of migraine with aura, and fully reversible motor weakness and/or visual, sensory and/ or speech/ language symptoms.

*Presenting neurological symptom* was recorded and also if there was any comorbidities and events that may have acted as a *precipitating factor* of disease onset.

*Motor delay*: when motor milestones occurred outside the 95% confidence interval, according to WHO.[^1^](#_ENREF_1)

*Intellectual disability:* intellectual difficulties, as well as difficulties in conceptual, social, and practical areas of living.[^2^](#_ENREF_2)

*Disability milestones:* gastrostomy, agraphia, falls, unilateral gait assistance, and confinement to wheelchair.

*Scale for the Assessment and Rating of Ataxia (SARA)* and *Inventory of Non-Ataxia signs (INAS)* were included at baseline and annual evaluations.

*MRI:* The report from the first brain MRI was reviewed for each patient (when available), and information was collected concerning atrophy in the following topographies: cerebellar vermis, cerebellar hemispheres, cerebellar peduncles, pons, mesencephalon, cerebral cortex and cervical spinal cord.

*Neurophysiological studies:* Abnormalities in nerve conduction studies were recorded, including presence of axonal/demyelinating neuropathy and sensitive/motor type.

**2. Workflow of genetic testing**

A) Single gene testing – 26 probands were studied. In 19 originating from local clusters and/or with a high clinical suspicion of MJD/SCA3, *ATXN3* was studied (15 with pathogenic expansion). In probands with a longstanding pure cerebellar syndrome *CACNA1A* expansion was studied (two patients, positive in one and negative in another). This last patient was subsequently tested for *PRKCG* with a pathogenic variant identified (before multigene WES-based panels were available). In probands with retinopathy, *ATXN7* was studied (one case, with pathogenic expansion). In cases with familial hemiplegic migraine, *CACNA1A* was studied (one case, with pathogenic variant). In a family with congenital ataxia *ITPR1* was studied (one case, previous to multigene panels). In two patients with childhood onset and no family history, *FXN* expansion was tested (negative results).

B) Multiplex repeat expansion – 21 probands were studied. 1) Four patients studied through single gene who did not carry an expansion in *ATXN3* were subsequently tested for the remaining polyQ ataxias. 2) Those who were from geographic areas with low prevalence of MJD, or who had no particular phenotypic trait which could guide testing for a particular form of ataxia (in particular no pure cerebellar syndrome, retinopathy nor hemiplegic migraine) were studied through multiplex repeat expansion (for *ATXN1, ATN1, ATXN2, ATXN3, CACNA1A, ATXN7,* *TBP*) *ab initium*. 3) Ten patients were not studied for polyQ expansions. In these, age at onset (occurring in childhood or early adolescence), phenotype and family history were highly unsuggestive of polyQ. Eight had non-cerebellar onset (congenital forms or paroxysmal episodes) and no known family history (either healthy parents or parents dying in the fourth-fifth decade of life without any neurological symptoms). Two, also without family history, had a cerebellar onset and had tested negative for *FXN* expansions.

C) Multigene WES-based panels – 28 probands were tested (one had been lost to follow up). 1) 18 patients who tested negative on multiplex repeat expansions (one had been lost to follow up), and 2) the 10 detailed above, whose clinical phenotype and family history was not suggestive of polyQ.

D) WES – 10 patients are currently under study

**3. Methodology of screening of ataxias caused by repeats’ expansion**

Spinocerebellar ataxias caused by repeats’ expansion (SCA1, SCA2, SCA3/MJD, SCA6, SCA7, SCA17 and DRPLA) were excluded by conventional methodologies. In brief, the genomic regions (*ATXN1, ATXN2, ATXN3, CACNA1A, ATXN7, TBP and ATN1 loci*) of the repeats were amplified by standard PCR conditions, using fluorescently-labelled primers. Products were subject to fragment analysis, using an ABI 3130xl genetic analyser (Applied Biosystems). Homoallelic cases (two normal alleles of the same size) were further tested by repeat-primed PCR (RP-PCR) followed by fragment analysis, to exclude the presence of a heterozygous large expansion.

**4. Whole-exome and NGS based panels**

Whole-exome sequencing (WES) was performed over the years using different capture kits (Agilent’s SureSelect Human All Exon V6 or Twist Comprehensive Exome including mitochondrial DNA) and sequencers ((Illumina’s HiSeq or NovaSeq 6000). For data analysis, a custom validated pipeline, based on the Broad Institute’s Best Practices, was applied, using bwa-mem for alignment to the GRCh37 build of the human genome, GATK HaplotypeCaller for variant calling, and Ensembl VEP and GEMINI for variant annotation. Quality control (QC) was performed on the resulting FASTQ (FastQC), BAM (QualiMap and samtools) and VCF (bcftools) files, aggregated on a QC report using MultiQC. Variant calling was initially restricted to a virtual panel of 236 nuclear genes and 37 mitochondrial genes (for hereditary cerebellar ataxias), and subsequently expanded to 1,411 genes (neuroexome) (gene lists below).

Variants were filtered by their minor allele-frequency (MAF below 1% in population databases: NCBI’s dbSNP, 1,000 Genome Project, Exome Variant Project, ExAC and gnomAD), for those that resulted in a change at the protein level and/or previously described in the Human Gene Mutation Database or NCBI’s ClinVar. All filtered variants were further analysed, using Alamut Visual (Interactive Biosoftware) for in-silico prediction of pathogenicity (including SIFT, PolyPhen2, CADD and splice-site prediction), population frequency confirmation and variants’ visual inspection in the alignment file. Sanger sequencing was used to confirm variants identified by WES and for segregation analysis.

**4.1 Virtual gene panel – WES-based multigene panel for hereditary ataxia (273 genes):**

*AARS2, ABCB7, ABHD12, ACO2, ADGRG1, ADPRS, AFG3L2, AHI1, AIFM1, ALG1, ALG11, ALG12, ALG13, ALG2, ALG3, ALG6, ALG8, ALG9, AMACR, ANO10, APTX, ARL13B, ARSA, ATAD3A, ATCAY, ATG5, ATG7, ATM, ATP13A2, ATP1A3, ATP2B3, ATP5MK, ATP6V0A2, ATP8A2, AUH, B4GALNT1, BCAP31, BEAN1, BRAT1, BTD, C19ORF12, CA8, CACNA1A, CACNA1G, CACNB4, CAMTA1, CAPN1, CASK, CC2D2A, CCDC88C, CEP290, CEP41, CHMP1A, CHP1, CLCN2, CLN5, CLN6, CLN8, CLPB, COA7, COQ2, COQ8A, COQ9, COX20, CP, CPLANE1, CSF1R, CSPP1, CWF19L1, CYP27A1, DARS2, DDOST, DLAT, DNAJC19, DNAJC3, DNAJC5, DNMT1, DOLK, DPAGT1, DPM1, DPM2, DPM3, EBF3, EEF2, EIF2B1, EIF2B2, EIF2B3, EIF2B4, EIF2B5, ELOVL4, ELOVL5, EPM2A, ERCC2, ERCC3, ERCC4, ERCC5, EXOSC3, FAT2, FGF14, FLVCR1, GBA2, GBE1, GCLC, GDAP2, GFAP, GJC2, GOSR2, GRID2, GRM1, IFRD1, INPP5E, IRF2BPL, ITPR1, KCNA1, KCNA2, KCNC3, KCND3, KCNJ10, KCNMA1, KIF1C, KIF5A, LAMA1, LYRM7, LYST, MARS2, MECR, MME, MPDU1, MPI, MRE11, MSTO1, MTPAP, MTTP, NDUFV1, NEU1, NHLRC1, NKX2-1, NKX6-2, NPC1, NPC2, NPHP1, NUS1, OPA1, PCNA, PDSS1, PDSS2, PDYN, PEX10, PEX2, PEX7, PGM1, PHYH, PIGS, PIK3R5, PLA2G6, PLD3, PMM2, PMPCA, PMPCB, PNKP, PNPLA6, POLG, POLR3A, PRDX3, PRICKLE1, PRKCG, PRPS1, PTRH2, PUM1, RARS2, RFT1, RNF170, RNF216, RPGRIP1L, RUBCN, SACS, SAMD9L, SCN1A, SCN2A, SCN8A, SCYL1, SETX, SIL1, SLC17A5, SLC1A3, SLC25A46, SLC52A2, SLC9A1, SNX14, SPG7, SPR, SPTBN2, SRD5A3, SSR4, STT3A, STT3B, STUB1, SURF1, SYNE1, SYT14, TCTN1, TDP1, TDP2, TGM6, THG1L, TMEM138, TMEM216, TMEM237, TMEM240, TMEM67, TPP1, TRPC3, TSEN2, TSEN34, TSEN54, TTBK2, TTC19, TTPA, TUBB4A, TWNK, UBA5, VAMP1, VLDLR, VPS13D, VPS41, VRK1, VWA3B, WARS2, WDR73, WDR81, WFS1, WWOX, XPA, XRCC1, ZFYVE26, ZNF59.*

*MT-ATP6, MT-ATP8, MT-CO1, MT-CO2, MT-CO3, MT-CYB, MT-ND1, MT-ND2, MT-ND3, MT-ND4, MT-ND4L, MT-ND5, MT-ND6, MT-RNR1, MT-RNR2, MT-TA, MT-TC, MT-TD, MT-TE, MT-TF, MT-TG, MT-TH, MT-TI, MT-TK, MT-TL1, MT-TL2, MT-TM, MT-TN, MT-TP, MT-TQ, MT-TR, MT-TS1, MT-TS2, MT-TT, MT-TV, MT-TW, MT-TY*

**4.2 Virtual gene panel – Neuroexome (1411 genes) (genes associated with neurological disorders):**

*AAAS, AARS1, AARS2, AASS, ABAT, ABCA1, ABCB7, ABCC6, ABCC8, ABCD1, ABCD4, ABHD12, ABHD5, ACACA, ACAD9, ACADM, ACADS, ACADSB, ACADVL, ACAT1, ACO2, ACOX1, ACSF3, ACSL4, ACTA1, ACTA2, ACTB, ACTG1, ACTG2, ACVRL1, ACY1, ADAMTS2, ADAR, ADAT3, ADCY5, ADD3, ADGRG1, ADGRV1, ADK, ADNP, ADRA2B, ADSL, AFF2, AFG3L2, AGA, AGK, AGL, AGPAT2, AGRN, AGXT, AHI1, AIFM1, AIMP1, AKT1, AKT3, ALAD, ALDH18A1, ALDH3A2, ALDH4A1, ALDH5A1, ALDH7A1, ALDOA, ALDOB, ALG1, ALG11, ALG12, ALG13, ALG14, ALG2, ALG3, ALG6, ALG8, ALG9, ALMS1, ALS2, ALX1, ALX3, ALX4, AMACR, AMN, AMPD1, AMPD2, AMT, ANG, ANK3, ANKLE2, ANKRD11, ANO10, ANO3, ANO5, ANTXR1, AP1S1, AP1S2, AP3B1, AP3B2, AP4B1, AP4E1, AP4M1, AP4S1, AP5Z1, APOA1, ApoE, APP, APTX, AR, ARFGEF2, ARG1, ARHGAP31, ARHGEF10, ARHGEF6, ARHGEF9, ARID1A, ARID1B, ARL13B, ARL6, ARL6IP1, ARSA, ARSB, ARSI, ARSL, ARV1, ARX, ASAH1, ASCL1, ASL, ASNS, ASPA, ASPM, ASS1, ASXL1, ASXL3, ATCAY, ATIC, ATL1, ATL3, ATM, ATN1, ATP13A2, ATP1A2, ATP1A3, ATP2A1, ATP2A2, ATP2B3, ATP5F1A, ATP5F1E, ATP6AP2, ATP6V0A2, ATP7A, ATP7B, ATP8A2, ATPAF2, ATR, ATRX, ATXN1, ATXN10, ATXN2, ATXN3, ATXN7, AUH, AUTS2, AVP, B3GALNT2, B3GLCT, B4GALNT1, B4GALT1, B4GAT1, B9D1, B9D2, BAG3, BBIP1, BBS1, BBS10, BBS12, BBS2, BBS4, BBS5, BBS7, BBS9, BCAP31, BCKDHA, BCKDHB, BCKDK, BCOR, BCS1L, BDNF, BEAN1, BICD2, BIN1, BOLA3, BRAF, BRAT1, BRF1, BRWD3, BSCL2, BSND, BVES, C12orf57, C12ORF65, C19ORF12, C9ORF72, CA8, CACNA1A, CACNA1B, CACNA1C, CACNA1D, CACNA1G, CACNA1S, CACNB4, CACNG2, CAD, CAMTA1, CAPN1, CAPN3, CARS2, CASK, CASQ1, CASR, CAV1, CAV3, CAVIN1, CBL, CC2D1A, CC2D2A, CCDC115, CCDC174, CCDC78, CCDC88C, CCM2, CCND2, CCT5, CD59, CD96, CDH15, CDK13, CDK5, CDK5RAP2, CDK6, CDKL5, CDON, CENPE, CENPJ, CEP104, CEP135, CEP152, CEP290, CEP41, CEP63, CERS1, CERT1, CFL2, CHAMP1, CHAT, CHCHD10, CHCHD2, CHD2, CHD7, CHKB, CHMP1A, CHMP2B, CHRNA1, CHRNA2, CHRNA4, CHRNA7, CHRNB1, CHRNB2, CHRND, CHRNE, CHRNG, CILK1, CIZ1, CLCN1, CLCN2, CLCN4, CLCNKA, CLCNKB, CLIC2, CLN3, CLN5, CLN6, CLN8, CLP1, CLPB, CNBP, CNNM2, CNTN1, CNTN2, CNTNAP2, COA5, COA6, COASY, COG1, COG4, COG5, COG6, COG7, COG8, COL11A2, COL12A1, COL13A1, COL18A1, COL4A1, COL4A2, COL6A1, COL6A2, COL6A3, COL9A3, COLQ, COQ2, COQ4, COQ6, COQ8A, COQ9, COX10, COX14, COX15, COX6A1, COX6B1, COX8A, CP, CPA6, CPLANE1, CPS1, CPT1C, CPT2, CRADD, CRB2, CRBN, CREBBP, CRIPT, CRPPA, CRYAB, CSF1R, CSNK2A1, CSPP1, CST3, CSTB, CTC1, CTCF, CTDP1, CTNNB1, CTSD, CTSF, CUL4B, CWF19L1, CYP26B1, CYP27A1, CYP2U1, CYP7B1, DAG1, DARS1, DARS2, DBT, DCAF17, DCAF8, DCTN1, DCX, DDHD1, DDHD2, DDOST, DDX3X, DEAF1, DEPDC5, DES, DGUOK, DHCR7, DHH, DHTKD1, DIAPH1, DIAPH3, DIP2B, DKC1, DLD, DLG3, DLL4, DMD, DMPK, DMXL2, DNAJB2, DNAJB6, DNAJC19, DNAJC3, DNAJC5, DNAJC6, DNM1, DNM1L, DNM2, DNMT1, DOCK6, DOCK7, DOK7, DOLK, DPAGT1, DPH1, DPM1, DPM2, DPM3, DPP6, DRD2, DST, DYNC1H1, DYNC2H1, DYRK1A, DYSF, EARS2, EBF3, EBP, ECEL1, EDC3, EEF1A2, EEF2, EFHC1, EFTUD2, EGR2, EHMT1, EIF2B1, EIF2B2, EIF2B3, EIF2B4, EIF2B5, EIF2S3, EIF4G1, ELAC2, ELOVL4, ELOVL5, ELP1, ELP2, EMC1, EMD, EMX2, ENO3, ENTPD1, EOGT, EP300, EPB41L1, EPM2A, ERBB4, ERCC1, ERCC2, ERCC5, ERCC6, ERLIN1, ERLIN2, ERMARD, ESCO2, ETFA, ETFB, ETFDH, ETHE1, EXOC8, EXOSC3, EXOSC8, EXT2, FA2H, FADD, FAM126A, FARS2, FASTKD2, FBLN5, FBN1, FBXL4, FBXO31, FBXO38, FBXO7, FGD1, FGD4, FGF12, FGF14, FGFR1, FGFR2, FH, FHL1, FIG4, FKRP, FKTN, FLNA, FLNC, FLVCR1, FLVCR2, FMN2, FMR1, FOLR1, FOXG1, FOXP1, FOXP2, FOXRED1, FREM1, FRMD4A, FRMD7, FRMPD4, FRRS1L, FTL, FTO, FTSJ1, FUS, FXN, G6PC1, GAA, GABRA1, GABRB1, GABRB3, GABRG2, GAD1, GAL, GALC, GALNS, GAMT, GAN, GARS1, GATAD2B, GATM, GBA, GBA2, GBE1, GCDH, GCH1, GCSH, GDAP1, GDI1, GDNF, GFAP, GFER, GFM1, GFPT1, GJB1, GJC2, GK, GLA, GLB1, GLDC, GLE1, GLI2, GLI3, GLRA1, GLRB, GLRX5, GM2A, GMPPA, GMPPB, GNAL, GNAO1, GNB1, GNB4, GNB5, GNE, GNPAT, GNS, GOSR2, GPC3, GPR143, GPT2, GRIA3, GRID2, GRIK2, GRIN1, GRIN2A, GRIN2B, GRIP1, GRM1, GRN, GSN, GTPBP3, GUF1, GUSB, GYG1, GYS1, GYS2, HACE1, HADHA, HADHB, HARS1, HCCS, HCFC1, HCN1, HDAC6, HDAC8, HECW2, HEPACAM, HERC1, HERC2, HESX1, HEXA, HEXB, HGSNAT, HIKESHI, HINT1, HIVEP2, HK1, HMGB3, HNMT, HNRNPA1, hnRNPA2B1, HNRNPDL, HNRNPH2, HOXA1, HOXD10, HPCA, HPRT1, HRAS, HSD17B10, HSD17B4, HSPB1, HSPB3, HSPB8, HSPD1, HSPG2, HTRA1, HTRA2, HTT, HUWE1, IARS1, IARS2, IBA57, IDS, IDUA, IER3IP1, IFIH1, IFT140, IFT27, IGBP1, IGF1, IGHMBP2, IKBKG, IL11RA, IL1RAPL1, IL1RN, INF2, INPP5E, INS, IQSEC2, IRX5, ISCU, ITGA7, ITM2B, ITPA, ITPR1, JAG1, JPH3, JRK, KANK1, KARS1, KAT6A, KAT6B, KATNB1, KBTBD13, KCNA1, KCNA2, KCNB1, KCNC1, KCNC3, KCND3, KCNJ1, KCNJ10, KCNJ11, KCNJ2, KCNK18, KCNK9, KCNMA1, KCNQ2, KCNQ3, KCNT1, KCTD17, KCTD7, KDM1A, KDM5C, KDM6A, KIAA0586, KIDINS220, KIF11, KIF1A, KIF1B, KIF1C, KIF21A, KIF2A, KIF4A, KIF5A, KIF5C, KIF7, KIRREL3, KLC2, KLHL15, KLHL40, KLHL41, KMT2A, KMT2B, KMT2D, KNL1, KPTN, KRAS, KRIT1, L1CAM, LAMA2, LAMB1, LAMB2, LAMC3, LAMP2, LARGE1, LBR, LDB3, LDHA, LGI1, LIG4, LIMS2, LINS1, LITAF, LMAN2L, LMBRD1, LMNA, LMNB1, LMNB2, LMOD3, LPIN1, LRP2, LRP4, LRPPRC, LRRK2, LRSAM1, LYRM4, LZTFL1, LZTR1, MAG, MAGEL2, MAN1B1, MAOA, MAPK10, MAPT, MARS1, MARS2, MASP1, MATR3, MBD5, MBTPS2, MCPH1, MECP2, MED12, MED13L, MED17, MED23, MED25, MEF2C, METTL23, MFF, MFN2, MFSD2A, MFSD8, MGAT2, MICU1, MID1, MID2, MKKS, MKS1, MLC1, MMAA, MMAB, MMACHC, MMADHC, MMUT, MOGS, MPDU1, MPDZ, MPI, MPV17, MPZ, MRE11, MRPL3, MRPL44, MRPS16, MRPS22, MSMO1, MSX2, MTFMT, MTHFR, MTM1, MTMR2, MTO1, MTOR, MTPAP, MTR, MTRR, MTTP, MUSK, MVK, MYBPC1, MYCN, MYF6, MYH14, MYH2, MYH3, MYH7, MYO5A, MYOT, MYT1L, NAA10, NAGA, NAGLU, NALCN, NARS2, NAT8L, NDE1, NDN, NDP, NDRG1, NDST1, NDUFA1, NDUFA10, NDUFA11, NDUFA12, NDUFA2, NDUFA4, NDUFA9, NDUFAF1, NDUFAF2, NDUFAF3, NDUFAF4, NDUFAF5, NDUFAF6, NDUFB3, NDUFB9, NDUFS1, NDUFS2, NDUFS3, NDUFS4, NDUFS6, NDUFS7, NDUFS8, NDUFV1, NDUFV2, NEB, NECAP1, NEDD4L, NEFH, NEFL, NEU1, NEXMIF, NF1, NF2, NFIX, NFU1, NGF, NHEJ1, NHLRC1, NHP2, NHS, NIPA1, NIPBL, NKX2-1, NLRP3, NME1, NOL3, NONO, NOP10, NOP56, NOTCH1, NOTCH2, NOTCH3, NPC1, NPC2, NPHP1, NPHP3, NPRL2, NPRL3, NRAS, NRXN1, NSD1, NSDHL, NSUN2, NT5C2, NTRK1, NTRK2, NUBPL, OCLN, OCRL, OFD1, OPA1, OPA3, OPHN1, OPTN, ORC1, OTC, PAFAH1B1, PAH, PAK3, PANK2, PARK7, PARN, PAX2, PAX3, PC, PCBD1, PCDH19, PCLO, PCNA, PCNT, PDCD10, PDE10A, PDE4D, PDE6D, PDE8B, PDGFB, PDGFRB, PDHA1, PDK3, PDSS1, PDSS2, PDYN, PEX1, PEX10, PEX11B, PEX12, PEX13, PEX14, PEX16, PEX19, PEX2, PEX26, PEX3, PEX5, PEX6, PEX7, PFKM, PFN1, PGAM2, PGAP1, PGAP2, PGAP3, PGK1, PGM1, PHC1, PHF6, PHF8, PHKA1, PHKA2, PHOX2B, PHYH, PI4KA, PIEZO2, PIGA, PIGG, PIGL, PIGN, PIGO, PIGT, PIGV, PIGW, PIGY, PIK3CA, PIK3R2, PIK3R5, PINK1, PLA2G6, PLCB1, PLEC, PLEKHG2, PLEKHG5, PLK4, PLP1, PMM2, PMP22, PMPCA, PNKD, PNKP, PNPLA2, PNPLA6, PNPLA8, PNPO, PNPT1, POGZ, POLG, POLG2, POLR1C, POLR3A, POLR3B, POMGNT1, POMGNT2, POMK, POMT1, POMT2, PORCN, PPP1R15B, PPP2R1A, PPP2R2B, PPP2R5D, PPT1, PQBP1, PRDM8, PREPL, PRICKLE1, PRICKLE2, PRKCG, PRKDC, PRKN, PRKRA, PRNP, PROP1, PRPS1, PRRT2, PRRX1, PRSS12, PRSS56, PRX, PSAP, PSEN1, PSEN2, PTCH1, PTEN, PTF1A, PTPN11, PTRH2, PTS, PURA, PUS1, PUS3, PYCR2, PYGL, PYGM, QARS1, QDPR, RAB18, RAB39B, RAB3GAP1, RAB3GAP2, RAB7A, RAD21, RAD50, RAF1, RAI1, RAPSN, RARB, RARS1, RARS2, RAX, RBBP8, RBM10, RBM28, RBM8A, RBP4, RBPJ, REEP1, REEP2, RELN, RERE, RETREG1, RFT1, RIN2, RIT1, RLIM, RMND1, RNASEH2A, RNASEH2B, RNASEH2C, RNASET2, RNF135, RNF170, RNF216, RNU4ATAC, ROGDI, ROR2, RPGRIP1L, RPL10, RPS6KA3, RRM2B, RTEL1, RTN2, RTN4IP1, RTTN, RUBCN, RXYLT1, RYR1, SACS, SAMHD1, SASS6, SBDS, SBF1, SBF2, SCARB2, SCN10A, SCN11A, SCN1A, SCN1B, SCN2A, SCN4A, SCN8A, SCN9A, SCO1, SCO2, SCP2, SCYL1, SDCCAG8, SDHA, SDHAF1, SELENON, SEPSECS, SEPTIN9, SERAC1, SERPINI1, SETBP1, SETD2, SETD5, SETX, SFXN4, SGCA, SGCB, SGCD, SGCE, SGCG, SGSH, SH3TC2, SHANK2, SHANK3, SHH, SHOC2, SHROOM4, SIGMAR1, SIK1, SIL1, SIX3, SKI, SLC12A1, SLC12A5, SLC12A6, SLC13A5, SLC16A2, SLC17A5, SLC19A3, SLC1A1, SLC1A2, SLC1A3, SLC1A4, SLC20A2, SLC22A5, SLC25A12, SLC25A19, SLC25A20, SLC25A22, SLC25A3, SLC25A4, SLC25A46, SLC2A1, SLC2A2, SLC30A10, SLC33A1, SLC35A1, SLC35A2, SLC35A3, SLC35C1, SLC37A4, SLC39A14, SLC39A8, SLC4A4, SLC5A7, SLC6A1, SLC6A17, SLC6A3, SLC6A5, SLC6A8, SLC9A6, SMARCA2, SMARCA4, SMARCB1, SMARCE1, SMC1A, SMC3, SMCHD1, SMN1, SMPD1, SMS, SNAP29, SNCA, SNCB, SNIP1, SNRPB, SNX14, SOBP, SOD1, SOS1, SOS2, SOX10, SOX11, SOX3, SPART, SPAST, SPATA5, SPG11, SPG21, SPG7, SPR, SPTAN1, SPTBN2, SPTLC1, SPTLC2, SQSTM1, SRD5A3, SRPX2, SSR4, ST3GAL3, ST3GAL5, STAC3, STAMBP, STIL, STRADA, STT3A, STT3B, STUB1, STX1B, STXBP1, SUCLA2, SUCLG1, SUFU, SUMF1, SURF1, SYN1, SYNE1, SYNE2, SYNGAP1, SYNJ1, SYP, SYT14, SYT2, SZT2, TACO1, TAF1, TAF2, TARDBP, TARS2, TAZ, TBC1D24, TBC1D7, TBCD, TBCE, TBCK, TBK1, TBL1XR1, TBP, TBX1, TCAP, TCF12, TCF4, TCTN1, TCTN2, TCTN3, TDGF1, TDP1, TDP2, TECPR2, TECR, TENM4, TFAM, TFAP2A, TFAP2B, TFG, TG, TGFB1, TGFB2, TGFB3, TGFBR1, TGFBR2, TGIF1, TGM6, TH, THAP1, THOC2, TIMM8A, TINF2, TK2, TKT, TMCO1, TMEM126A, TMEM138, TMEM165, TMEM199, TMEM216, TMEM231, TMEM237, TMEM240, TMEM43, TMEM67, TMEM70, TMLHE, TNIK, TNNI2, TNNT1, TNNT3, TNPO3, TOR1A, TOR1AIP1, TPK1, TPM2, TPM3, TPP1, TRAPPC11, TRAPPC9, TRDN, TREM2, TREX1, TRIM2, TRIM32, TRIO, TRMT10A, TRNT1, TRPM6, TRPS1, TRPV4, TSC1, TSC2, TSEN15, TSEN2, TSEN34, TSEN54, TSFM, TSHB, TSPAN7, TTBK2, TTC19, TTC21B, TTC8, TTI2, TTN, TTPA, TTR, TUBA1A, TUBA4A, TUBA8, TUBB, TUBB2A, TUBB2B, TUBB3, TUBB4A, TUBG1, TUBGCP4, TUBGCP6, TUFM, TUSC3, TWNK, TXN2, TYMP, TYROBP, UBA1, UBA5, UBE2A, UBE3A, UBQLN2, UCHL1, UMPS, UNC80, UPB1, UPF3B, UQCC2, UQCC3, UQCRB, UQCRQ, USP27X, USP8, USP9X, VAMP1, VANGL1, VAPB, VARS2, VAX1, VCP, VIPAS39, VLDLR, VMA21, VPS11, VPS13A, VPS13B, VPS13C, VPS33B, VPS35, VPS37A, VPS53, VRK1, VWA3B, WAC, WASHC4, WASHC5, WDPCP, WDR45, WDR48, WDR62, WDR81, WFS1, WNK1, WNT3, WNT5A, WNT7A, WRAP53, WWOX, XK, XPA, XPC, XPR1, XRCC4, XYLT1, YAP1, YARS1, YARS2, ZBTB16, ZBTB18, ZC3H14, ZDHHC15, ZDHHC9, ZEB2, ZFR, ZFYVE26, ZFYVE27, ZIC1, ZIC2, ZMYND11, ZNF335, ZNF423, ZNF592, ZNF711.*

**5. Whole-exome sequencing**

Exome sequencing libraries were prepared using the SureSelect Exome Capture Kit v7 (Agilent), and sequencing was performed on NextSeq550 (Illumina), with 100 bp paired-end reads, according to manufacturer’s instructions. After sequencing, reads were aligned to the human reference genome hg19/GRCh37, using Burrows-Wheeler Aligner (BWA) v0.7.1, and variants called using GATK best practices v3.3-0. Duplicate reads were identified through samblaster v0.1.21. Variant annotation was performed with snpEff v4.2 and dbNSFP v2.9. Annotated variants were filtered and prioritized using Exomiser v7.2.1 and based on several criteria, including: minor allele frequency, MAF, <1% in reference population databases (Nucleotide Polymorphism Database [dbSNP], Genome Aggregation Database [gnomAD v2.1], 1,000 Genome Project); correlation with the phenotype (human phenotype ontology HP:0001251 [term name:ataxia]); and functional impact (SIFT, Polyphen2, Mutation assessor, FATHMM and UMD-Predictor). Sanger sequencing was used to confirm variants identified by WES and for segregation analysis.

**References**

1. Group WHOMGRS. WHO Motor Development Study: windows of achievement for six gross motor development milestones. Acta Paediatr Suppl 2006;450:86-95.

2. American Psychiatric Association. (2013). Diagnostic and statistical manual of mental disorders (5th ed.). <https://doi.org/10.1176/appi.books.9780890425596>.
