# Supplementary material for: Spinocerebellar Ataxias: Phenotypic Spectrum of PolyQ versus Non-Repeat Expansion Forms
Source: Cerebellum. 2024 Jul 24;23(6):2258–68. doi: 10.1007/s12311-024-01723-9 (PMC11585503; doi:10.1007/s12311-024-01723-9)
Supplement: Supplementary file 2 — Supplementary Table 1 Median number of repeats in each allele in patients from the polyQ ataxia group. Supplementary Table 2 Genetic forms in polyQ and non-repeat expansion SCA. Supplementary Table 3 Detailed clinical characterization of patients with polyQ ataxias (individual data). Supplementary Table 4 Detailed clinical characterization of patients with non-repeat expansion SCA (individual data). Supplementary Table 5 Disease duration in years [Q1-Q3] until symptom and disability milestone ultimately reached, according to the type of ataxia (DOCX 56 kb) [file 12311_2024_1723_MOESM2_ESM.docx]

**Supplementary Table 1** – Median number of repeats in each allele in patients from the polyQ ataxia group

| SCA | Patients, total / Patients with available data, n (%) | Number of repeats in the normal allele, median [Q1-Q3] | Number of repeats in the expanded allele, median [Q1-Q3] |
| --- | --- | --- | --- |
| MJD (SCA3) | 22/29 (75.9%) | 23.0 [19.5-27.0] | 70.5 [67.8-75.3] |
| SCA2 | 10/10 (100%) | 22.0 [22.0-22.0] | 39.0 [36.8-45.0] |
| SCA6 | 1/1 (100%) | NA | >19* |
| SCA7 | 1/1 (100%) | 10 | 47 |

*NA – not available, * test performed at the patient home-country where the patient*

**Supplementary Table 2** – Genetic forms in polyQ and non-repeat expansion SCA

|  | Number (frequency) | |
| --- | --- | --- |
|  | Families | Patients |
| **PolyQ SCA** |  |  |
| MJD/ SCA3 | 14 (*73.7%*) | 27 (*71.1%*) |
| SCA2 | 3 (*15.8%*) | 9 (2*3.7%*) |
| SCA6 | 1 (*5.3%*) | 1 (2.6*%*) |
| SCA7 | 1 (*5.3%*) | 1 (2.6*%*) |
| **Non-repeat expansion SCA** |  |  |
| ATX-*CACNA1A* | 3 (*14.3%*) | 10 (27.8*%*) |
| *ATP1A3*-related ataxia | 2 (*9.5%*) | 4 (11.1*%*) |
| ATX-*ITPR1* | 2 (*9.5%*) | 3 (8.3*%*) |
| *ATX/HSP-KCNA2* | 2 (*9.5%*) | 3 (8.3*%*) |
| ATX-*PRKCG* | 2 (*9.5%*) | 2 (5.6*%*) |
| ATX-*KCNC3* | 1 (*4.8%*) | 4 (11.1*%*) |
| ATX- *TMEM24* | 1 (*4.8%*) | 2 (5.6*%*) |
| ATX-*ELOVL4* | 1 (*4.8%*) | 1 (2.8*%*) |
| ATX-*GRM1* | 1 (*4.8%*) | 1 (2.8*%*) |
| ATX *STUB1* | 1 (*4.8%*) | 1 (2.8*%*) |
| HSP-*SPAST* | 1 (*4.8%*) | 1 (2.8*%*) |
| *CTNNB1* | 1 (*4.8%*) | 1 (2.8*%*) |
| HSP-*KIF1A* | 1(*4.8%*) | 1 (2.8*%*) |
| DYT-*KMT2B* | 1(*4.8%*) | 1 (2.8*%*) |
| *SYNGAP1* | 1(*4.8%*) | 1 (2.8*%*) |

**Supplementary Table 3 -** Clinical characterization of patients with polyglutamine (polyQ) ataxias

| Family | Patient  (gender) | Disease/ gene | Norm. allele/  Exp. allele  (nr. repeats) | Age | | First symptom | Ataxia | Oculomotor apraxia | Ophthalmo-paresis | Pyramidal signs | Peripheral neuropathy | Movement disorders | Motor delay | ID | Parox.  ataxia | Parox. dystonia | Hemipl.  migraine | Epilepsy | Age | |
| --- | --- | --- | --- | --- | --- | --- | --- | --- | --- | --- | --- | --- | --- | --- | --- | --- | --- | --- | --- | --- |
|  |  |  |  | Onset | First obs. |  |  |  |  |  |  |  |  |  |  |  |  |  | One cane | Wheel-chair bound |
| A | 1 (F) | MJD/SCA3 *ATXN3* | 22/65 | 29 | 33 | Parkinsonism | N | N | N | Y | N | Parkinsonism  Segm. dystonia | N | N | N | N | N | N | - | - |
| B | 2 (F) | MJD/SCA3 *ATXN3* | 23/76 | 26 | 30 | Gait ataxia | Y | N | N | Y | N | Segm. dystonia | N | N | N | N | N | N | - | - |
|  | 3 (F) |  | Unk | 36 | 22 | Gait ataxia | Y | N | N | Y | N | Segm. dystonia | N | N | N | N | N | N | - | - |
|  | 6 (F) |  | Unk | 45 | 46 | Diplopia | Y | N | Y | Y | Y | Segm. dystonia | N | N | N | N | N | N | 59 | 62 |
|  | 7 (F) |  | Unk | 44 | 50 | Gait ataxia | Y | N | Y | N | Y | Segm. dystonia | N | N | N | N | N | N | - | 67 |
|  | 8 (F) |  | 27/67 | 57 | 58 | Gait ataxia | Y | N | Y | N | Y | Segm. dystonia | N | N | N | N | N | N | - | - |
|  | 9 (F) |  | 21/76 | 41 | 55 | Gait ataxia | Y | N | Y | Y | N | Segm. dystonia | N | N | N | N | N | N | 50 | 52 |
|  | 10 (F) |  | Unk/67 | 43 | 43 | UL dysmetria | Y | N | N | N | N | N | N | N | N | N | N | N | - | - |
|  | 11(M) |  | 23/70 | 51 | 53 | Diplopia | Y | N | N | Y | N | N | N | N | N | N | N | N | - | - |
|  | 12 (M) |  | 14/70 | 40 | 62 | Gait ataxia | Y | N | Y | Y | Y | Segm. dystonia | N | N | N | N | N | N | - | 67 |
|  | 13 (M) |  | 46/68 | 43 | 55 | Gait ataxia | Y | N | Y | N | Y | Focal dystonia | N | N | N | N | N | N | - | - |
|  | 14 (F) |  | 18/70 | 40 | 62 | Gait ataxia | Y | N | Y | N | Y | Segm. dystonia | N | N | N | N | N | N | 55 | - |
| C | 15 (M) | MJD/SCA3 *ATXN3* | 23/66 | 62 | 65 | Gait ataxia | Y | N | Y | N | Y | Tremor R/P  Parkinsonism | N | N | N | N | N | N | 72 | 73 |
| D | 16 (M) | MJD/SCA3 *ATXN3* | 29/66 | 59 | 68 | Gait ataxia | Y | N | Y | N | Y | Segm. dystonia | N | N | N | N | N | N | - | - |
| E | 17 (F) | MJD/SCA3 ATXN3 | 28/71 | 32 | 45 | Gait ataxia | Y | N | Y | N | Y | N | N | N | N | N | N | N | - | - |
|  | 18 (M) |  | 23/72 | 34 | 47 | Gait ataxia | Y | N | Y | N | N | Segm. dystonia | N | N | N | N | N | N | - | - |
| F | 19 (M) | MJD/SCA3 *ATXN3* | 18/76 | 40 | 41 | UL dysmetria | Y | N | N | Y | Y | Segm. dystonia | N | N | N | N | N | N | 52 | - |
|  | 20 (M) |  | 28/74 | 37 | 41 | Gait ataxia | Y | N | Y | Y | N | Segm. dystonia | N | N | N | N | N | N | 42 | - |
| G | 21 (M) | MJD/SCA3 *ATXN3* | 27/76 | 34 | 43 | Gait ataxia | Y | N | Y | Y | Y | Segm. dystonia | N | N | N | N | N | N | 41 | 42 |
| H | 22 (M) | MJD/SCA3 *ATXN3* | Unk | 24 | 24 | Nystagmus | Y | N | Y | Y | Y | Segm. dystonia | N | N | N | N | N | N | 45 | 46 |
|  | 23 (F) |  | 20/76 | 24 | 33 | Gait ataxia | Y | N | Y | Y | Y | Segm. dystonia | N | N | N | N | N | N | 36 | 40 |
| I | 24 (M) | MJD/SCA3 *ATXN3* | 23/73 | 34 | 34 | Gait ataxia | Y | N | N | Y | N | N | N | N | N | N | N | N | - | - |
| J | 25 (M) | MJD/SCA3 *ATXN3* | 26/71 | 38 | 51 | Gait ataxia | Y | N | Y | Y | N | Segm. dystonia | N | N | N | N | N | N | 45 | - |
| K | 26 (M) | MJD/SCA3 *ATXN3* | 17/73 | 33 | 37 | Gait ataxia | Y | N | N | Y | N | Segm. dystonia | N | N | N | N | N | N | - | - |
| L | 27 (F) | MJD/SCA3 *ATXN3* | 23/70 | 42 | 67 | Gait ataxia | Y | N | Y | Y | Y | N | N | N | N | N | N | N | - | 60 |
| M | 28 (F) | MJD/SCA3 *ATXN3* | Unk | 30 | 432 | Parkinsonism | Y | N | Y | Y | Y | Parkinsonism  Gen. dystonia  Tremor R | N | N | N | N | N | N | 47 | 48 |
| N | 29 (M) | MJD/SCA3 *ATXN3* | 14/70 | 39 | 41 | Gait ataxia | Y | N | Y | Y | N | N | N | N | N | N | N | N | - | - |
| O | 30 (M) | *SCA2/*  *ATXN2* | 22/39 | 30 | 41 | Gait ataxia | Y | Y | Y | N | Y | Segm. dystonia  Tremor R+P | N | N | N | N | N | N | 50 | 55 |
|  | 31 (F) |  | 26/39 | 70 | 74 | Gait ataxia | Y | N | N | N | N | N | N | N | N | N | N | N | 81 | - |
|  | 32 (F) |  | 22/41 | 21 | 29 | UL dysmetria | Y | N | N | N | Y | Segm. dystonia  Tremor K | N | N | N | N | N | N | 35 | 39 |
|  | 33 (M)* |  | 22/41 | 23 | 28 | Gait ataxia | Y | N | N | Y | N | Segm. dystonia  Tremor K  Chorea* | N | N | N | N | N | N | - | - |
|  | 34 (M) |  | 22/41 | 47 | 56 | Gait ataxia | Y | N | Y | Y | N | N | N | N | N | N | N | N | - | - |
|  | 25 (F)** |  | 22/39 | 28 | 29 | Gait ataxia | Y | N | N | Y | N | Segm. dystonia  Chorea** | N | N | N | N | N | N | - | 36 |
| P | 36 (M) | *SCA2/*  *ATXN2* | 22/37 | 59 | 62 | Gait ataxia | Y | N | N | Y | Y | Segm. dystonia  Parkinsonism | N | N | N | N | N | N | 70 | - |
|  | 38 (M) |  | 22/36 | 53 | 54 | Dysarthria | Y | N | N | N | N | N | N | N | N | N | N | N | - | - |
|  | 39 (F) |  | 22/35 | 55 | 62 | Gait ataxia | Y | N | Y | N | Y | Segm. dystonia | N | N | N | N | N | N | 73 | - |
| R | 40 (M) | *SCA6/*  *CACNA1A exp* | >19 | 40 | 50 | Gait ataxia | Y | N | N | N | N | N | N | N | N | N | N | N | 50 | - |
| S | 41 (F) | *SCA7/*  *ATXN7* | 10/47 | 22 | 25 | Retinopathy | Y | N | Y | Y | N | Segm. dystonia  Tremor P | N | N | N | N | N | N | 29 | . |

*Exp. – expanded; F – female; Gen. – Generalized; Hemipl- hemiplegic; ID – intellectual disability; Obs. – Observation; M – male; N – absent; Norm. – normal; Nr. – number; Parox. – paroxysmal; Segm. – segmental; Tremor P – Postural Tremor; Tremor R – Rest tremor; Tremor K – Kinetic tremor; UL – Upper limb; Wheelc – wheelchair; Y – present, * patient with concurrent Huntington Disease (18/50 CAG repeats); ** patient with concurrent Huntington Disease (18/52 CAG repeats).*

**Supplementary Table 4 -** Clinical characterization of non-repeat expansion ataxias

| Family | Patient (gender) | Gene | Variant | | Age | | First symptom | Ataxia | Oculomotor apraxia | Ophthalmoparesis | Pyramidal signs | Peripheral neuropathy | Movement disorders | Motor delay | ID | Parox. ataxia | Parox. dystonia | Hemipl. migraine | Epilepsy | Age | |
| --- | --- | --- | --- | --- | --- | --- | --- | --- | --- | --- | --- | --- | --- | --- | --- | --- | --- | --- | --- | --- | --- |
|  |  |  | cDNA | Protein | Onset | First obs. |  |  |  |  |  |  |  |  |  |  |  |  |  | One cane | Wheelc.  bound |
| A | 1 (M) | *ATP1A3* | c.2452G>A | p.(Glu818Lys) | 1 | 1 | Gait ataxia | Y | N | Y | N | Y | Segm. dystonia  chorea | Y | Y | Y | Y | N | N | N | N |
|  | 2 (F) |  |  |  | 2 | 3 | Gait ataxia | Y | Y | N | N | N | Gen. Dystonia  Chorea | N | N | N | N | N | N | N | N |
|  | 3 (F) |  |  |  | 1 | 48 | Gait ataxia | Y | N | N | N | N | Segm. dystonia  Chorea | N | N | N | N | N | N | N | N |
| B | 4 (M) | *ATP1A3* | c.374T>A | p.(Val125Glu) | 11 | 31 | Paroxysmal dystonia | Y | N | N | N | N | Gen. Dystonia | N | N | N | N | N | N | N | N |
| C | 5 (F) | *CACNA1A* | c.593G>A | p.Arg198Gln | 32 | 40 | Gait ataxia | Y | N | N | N | Y | Segm. dystonia | N | N | N | N | N | N | N | N |
| D | 6 (M) | *CACNA1A* | c.4996C>G | p.(Arg1666Gly) | 19 | 21 | Gait ataxia | Y | N | N | Y | N | N | N | N | N | N | N | N | N | N |
| E | 7 (M) | *CACNA1A* | c.1748 | p.583Q | 13 | 28 | Gait ataxia | Y | N | N | N | N | N | N | N | N | N | N | N | N | N |
|  | 8 (M) |  |  |  | 7 | 22 | Hemiplegic migraine | Y | N | N | N | N | Segm. dystonia | N | N | N | N | Y | N | N | N |
|  | 9 (F) |  |  |  | 10 | 23 | Hemiplegic migraine | Y | N | N | N | N | N | N | N | N | N | Y | N | N | N |
|  | 10 (F) |  |  |  | 35 | 45 | Gait ataxia | Y | N | N | N | N | N | N | N | N | N | N | N | N | N |
|  | 11 (F) |  |  |  | 7 | 46 | Hemiplegic migraine | Y | N | N | Y | N | N | N | N | N | N | Y | Y  Focal | N | N |
|  | 12 (F) |  |  |  | 15 | 29 | Hemiplegic migraine | Y | N | N | Y | N | N | N | N | N | N | Y | N | N | N |
|  | 13 (M) |  |  |  | 2 | 19 | Hemiplegic migraine | Y | N | N | Y | N | N | N | N | N | N | Y | N | N | N |
|  | 14 (F) |  |  |  | 14 | 32 | Hemiplegic migraine | Y | N | N | Y | N | N | N | N | N | N | Y | N | N | N |
| F | 15 (F) | *CTNNB1* | c.1543C>T | p.(Arg515*) | 0 | 2 | Motor delay | Y | Y | Y | Y | N | Gen. Dystonia | Y | Y | N | N | N | N | N | N |
| G | 16 (F) | *ELOVL4* | c.517A>T | p.(Ile191Phe) | 28 | 65 | Gait ataxia | Y | N | N | Y | N | Segm. dystonia | N | N | N | N | N | N | N | N |
| H | 17 (F) | *GRM1* | c.785A>G | p.(Tyr262Cys) | 50 | 57 | Gait ataxia | Y | N | N | Y | N | Segm. dystonia | N | N | N | N | N | N | 77 | N |
| I | 18 (F) | *ITPR1* | c.805C>T | p.(Arg269Trp) | 0 | 2 | Motor delay | Y | N | Y | Y | N | Segm. dystonia | Y | Y | N | N | N | N | 19 | N |
|  | 19 (F) |  |  |  | 0 | 59 | Motor delay | Y | N | Y | N | N | Segm. dystonia | Y | Y | N | N | N |  | 59 | N |
| J | 20 (F) | *ITPR1* | .11:g.(?_4490850)_(4836971_?) |  | 20 | 40 | UL dysmetria | Y | N | N | N | Y | N | N | N | N | N | N | N | 61 | N |
| K | 21 (F) | *KCNA2* | c.881G>A | p.(Arg294His) | 3 | 5 | Spastic gait | Y | N | N | Y | N | Segm. dystonia | N | Y | N | N | N | N | 27 | N |
| L | 22 (F) | *KCNA2* | c.881G>A | p.(Arg294His) | 3 | 3 | Epilepsy | Y | N | N | Y | N | Segm. dystonia | N | N | N | N | N | Y  Gen | N | N |
|  | 23 (M) |  |  |  | 1 | 35 | Epilepsy | Y | N | N | Y | N | Segm. dystonia | N | N | N | N | N | Y Gen | 45 | N |
| M | 24 (M) | *KCNC3* | c.1268G>A | p.(Arg423His) | 0 | 1 | Motor delay | Y | N | N | Y | N | Tremor P | Y | Y | N | N | N | N | N | N |
|  | 25 (F) |  |  |  | 0 | 15 | Motor delay | Y | N | N | N | N | Segm. dystonia | Y | Y | N | N | N | N | N | N |
|  | 26 (M) |  |  |  | 0 | 4 | Psychomotor delay | Y | N | N | Y | N | N | Y | Y | N | N | N | N | N | N |
|  | 27 (M) |  |  |  | 1,5 | 4 | Gait ataxia | Y | N | N | N | N | Tremor P | Y | Y | N | N | N | N | N | N |
| N | 28 (F) | *KIF1A* | c.761G>A | p.(Arg254Gln) | 5 | 26 | Gait ataxia | Y | N | N | Y | N | N | N | Y | N | N | N | N | 43 | N |
| O | 29 (F) | *KMT2B* | c.3334+1G>A | .(Gly1020_Asn1111del) | 22 | 25 | UL dysmetria | Y | N | Y | Y | Y | Segm. dystonia | N | N | N | N | N | Y  Focal | 30 | N |
| P | 30 (F) | *PRKCG* | c.1545del | p.(Cys516Alafs¨7) | 55 | 70 | Gait ataxia | Y | N | N | N | N | Segm. dystonia  Parkinsonism  Chorea | N | N | N | N | N | N | 70 | N |
| Q | 31 (M) | *PRKCG* | c.319A>G | p.(Ser107Gly) | 53 | 64 | UL dysmetria | Y | N | N | N | N | Segm. dystonia | N | N | N | N | N | N | N | N |
| R | 32 (F) | *SPAST* | c.1496G>A | p.(Arg499H) | 0 | 1 | Motor delay | Y | N | N | Y | Y | Segm. dystonia | Y | Y | N | N | N | N | N | 7 |
| S | 33 (F) | *STUB1* | c.734_735del | p.(Ile245Asnfs*23) | 36 | 45 | Chorea | Y | N | N | Y | N | Segm. dystonia  Chorea | N | N | N | N | N | N | N | N |
| T | 34 (F) | *SYNGAP1* | c.2063_2066delinsTGTATGCC | (p.(Glu688Valfs*53) | 0 | 3 | Motor delay | Y | N | N | Y | N | Gen. Dystonia | Y | Y | N | N | N | Y | N | N |
| U | 35 (F) | *TMEM240* | c.486_487del | (Tyr163Profs*69) | 22 | 35 | Gait ataxia | Y | N | N | Y | N | Segm. dystonia  Tremor P | N | N | N | N | N | N | N | N |
|  | 36 (F) |  |  |  | 15 | 68 | Gait ataxia | Y | N | N | Y | N | N | N | N | N | N | Y | N | 66 | 77 |

*F – female; M – male; Hemipl- hemiplegic; ID – intellectual disability; Obs. – Observation; Y – present; N – absent; Norm. – normal; Parox. – paroxysmal; Segm. – segmental; Tremor P – Postural tremor; Tremor R – Rest tremor; Tremor K – Kinetic tremor; UL – Upper limb*

**Supplementary Table 5** – Disease duration in years [Q1-Q3] until symptom and disability milestone ultimately reached, according to the type of ataxia

|  | **Polyglutamine**  **ataxias (n=38)** | **Non-repeat expansion ataxias (n=36)** | **P value** |
| --- | --- | --- | --- |
| Gait unsteadiness | 0.0 y *[0.0-1.0]* | 2.0 y [*0.0-15.0*] | **<0.001** |
| Upper limb dysmetria | 5.0 y [*1.8-10.2*] | 9.0 y [*2.0-20.0*] | 0.097 |
| Dysarthria | 6.5 y [*2.0-16.0*] | 6.0 y [*2.0-16.0*] | 0.761 |
|  |  |  |  |
| Diplopia | 5.5 y [*1.8-13.2*] | 29.5 y [*13.5-50.8*] | **0.010** |
| Dystonia | 8.0 y [*4.0-15.2*] | 8.0 y [*0.0-23.0*] | 0.527 |
| Tremor | 5.0 y [*3.0-8.0*] | 11.2 y [*4.6-39.2*] | 0.181 |
| Chorea | 4.0 y [*1.0--*] | 10.0 y [*2.5-27.0*] | 0.439 |
| Parkinsonism | 2.5 y [*0.0-8.8*] | 2.5 y [*0.0-7.5*] | 0.147 |
| Myoclonus | - | 5.0 y [*2.5-7.5*] | NA |
| Dysphagia | 11.0 y [*5.0-16.0*] | 26.0 y [*17.5-38.0*] | **<0.001** |
|  |  |  |  |
| Neuropathy | 14.0 y [*8.0-22.0*] | 11.0 y [*2.0-25.0*] | 0.668 |
| Seizures | - | 2.0 y [*0.0-4.7*] | NA |
|  |  |  |  |
| Depression | 7.0 y [*3.7-13.5*] | 12.0 y [*2.2-20.7*] | 0.297 |
| Anxiety | 7.5 y [*3.2-12.5*] | 15.0 y [*1.5-23.5*] | 0.152 |
| Psychosis | - | 17.0 y | NA |
|  |  |  |  |
|  |  |  |  |
| Agraphia | 15.0 y [*14.0-20.0*] | 21.0 y [*7.0--*] | 1.000 |
| Falls | 10.0 y [*6.5-14.0*] | 7.5 y [*2.7-24.0*] | 0.835 |
| Unilateral assistance in gait | 11.5 y [*8.5-15.5*] | 32.5 y [*18.0-45.7*] | **<0.001** |
| Confinement to wheelchair | 18.0 y [*11.0-22.0*] | 34.5 y [*7.00- -*] | 1.000 |

*NA – non applicable, Y - years*
